# Seasonality of the transmissibility of hand, foot and mouth disease: a modelling study in Xiamen City, China

**DOI:** 10.1017/S0950268819002139

**Published:** 2019-12-30

**Authors:** Zehong Huang, Mingzhai Wang, Luxia Qiu, Ning Wang, Zeyu Zhao, Jia Rui, Yao Wang, Xingchun Liu, Mikah Ngwanguong Hannah, Benhua Zhao, Yanhua Su, Bin Zhao, Tianmu Chen

**Affiliations:** 1 State Key Laboratory of Molecular Vaccinology and Molecular Diagnostics, School of Public Health, Xiamen University, Xiamen City, Fujian Province, People's Republic of China; 2 Xiamen Centre for Disease Control and Prevention, Xiamen City, Fujian Province, People's Republic of China; 3 Respiratory Department, Shanghai General Hospital, Shanghai, People's Republic of China; 4 Medical College, Xiamen University, Xiamen City, Fujian Province, People's Republic of China; 5 State Key Laboratory of Molecular Vaccinology and Molecular Diagnostics, Laboratory Department, Xiang'an Hospital of Xiamen University, Xiamen City, Fujian Province, People's Republic of China

**Keywords:** Effective reproduction number, hand, foot and mouth disease, seasonality, transmissibility

## Abstract

This study attempts to figure out the seasonality of the transmissibility of hand, foot and mouth disease (HFMD). A mathematical model was established to calculate the transmissibility based on the reported data for HFMD in Xiamen City, China from 2014 to 2018. The transmissibility was measured by effective reproduction number (*R*_eff_) in order to evaluate the seasonal characteristics of HFMD. A total of 43 659 HFMD cases were reported in Xiamen, for the period 2014 to 2018. The median of annual incidence was 221.87 per 100 000 persons (range: 167.98/100,000–283.34/100 000). The reported data had a great fitting effect with the model (*R*^2^ = 0.9212, *P* < 0.0001), it has been shown that there are two epidemic peaks of HFMD in Xiamen every year. Both incidence and effective reproduction number had seasonal characteristics. The peak of incidence, 1–2 months later than the effective reproduction number, occurred in Summer and Autumn, that is, June and October each year. Both the incidence and transmissibility of HFMD have obvious seasonal characteristics, and two annual epidemic peaks as well. The peak of incidence is 1–2 months later than *R*_eff_.

## Introduction

Hand, foot and mouth disease (HFMD), resulting from a group of enterovirus (mainly include enterovirus 71 (EV-A71) and coxsackievirus A16 (CV-A16) [1]), is an infectious disease common in children under 5 years of age. Clinical manifestations of HFMD are characterised by self-limited fever, typical skin rash, with or without mouth ulcers, complications of the nervous or cardiopulmonary systems which can also be found in several cases, and even results in death sometimes [2].

HFMD has spread widely in the Western Pacific Region [3]. Millions of infections were reported to have caused 96 900 Disability Adjusted of Life Years in some Asian countries [4], and had led to an extremely heavy disease burden in Asia and to the world at large.

The significance of prevention and control of HFMD is particularly important in China. More than 2.4 million infections are reported in China per year [3], with the enormous population base resulting in severe complications and even deaths. The outbreak of HFMD occurs almost every year [5]. All of the above make HFMD into a non-negligible emergency.

Since the first report in Shanghai, 1981, varying degrees of outbreaks have taken place in Dandong, Wuhan, Hong Kong, Taiwan etc. [5], which has brought bitter suffering to the state. Until May 2008, HFMD was defined as a C-class disease in Mainland China, which opened its surveillance and brought a new stage for the study of anti-HFMD measures.

Finding the potential factors affecting transmissibility of HFMD and establishing a suitable HFMD transmission model to predict the epidemic trend will undoubtedly bring important clues to the prevention and control of HFMD [6]. In recent years, several mathematical models have been developed to calculate the transmissibility of HFMD including spatial factors [7], climatic factors [8] and even environmental and socioeconomic factors [9], which have a great effect in predicting its epidemic trend. But there are still some deficiencies among the studies of seasonal transmissibility of HFMD. The HFMD has obvious seasonality, which is in the cross of spring and summer, summer and autumn and the seasonal fluctuation increases as the location approaches the equator [10]. The seasonal study of HFMD can provide an effective basis for preventing and controlling HFMD.

Therefore, this study was based on the susceptible-exposed-infectious-asymptomatic-removed (SEIAR) model, combined with the epidemiological characteristics of HFMD transmission, and established a SEIAR dynamic model of HFMD epidemic with seasonality. The daily incidence data from 2014 to 2018 in Xiamen were collected to evaluate the seasonal transmissibility of HFMD, so as to provide clues for the development of disease prevention and control measures.

## Materials and methods

### Study design

In this study, the mathematical epidemiological method was used to mine the data by the SEIAR model in order to master the characteristics and transmissibility of HFMD.

### Date collection

Xiamen, a large city in Fujian Province, is located on the south-east coast of China and belongs to the subtropical monsoon climate area. According to the 2018 census, the resident population of Xiamen has exceeded 4 million. The data of reported HFMD cases from Xiamen were extracted from the China Information System for Disease Control and Prevention from January 2014 to December 2018. All cases included were diagnosed in accordance with the *Hand*, *Foot and Mouth Disease Diagnosis and Treatment Guide* (*2010 Edition*) and *Diagnosis for Hand*, *Foot and Mouth Disease* (*WS*-588 2018), issued by the National Health Commission of the People's Republic of China.

### Transmission model

**Fig. 1.**
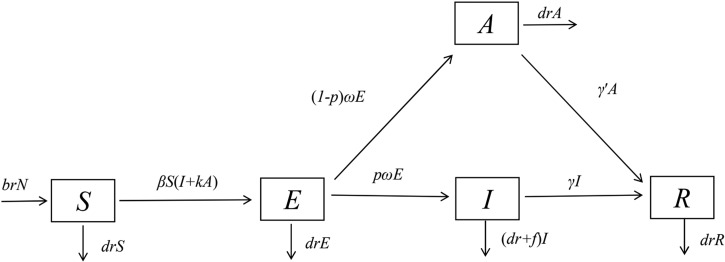
Flow chart of the SEIAR model for HFMD.

Our previous study shows that the susceptible-infectious-removed model could be used to calculate the transmissibility of HFMD based on weekly incidence data [11]. In this study, daily incidence data were employed. Therefore, the incubation period and asymptomatic infection were considered. Thus, a SEIAR model [11, 12] was established in this study. According to the natural history of HFMD [3, 13], the population was divided into susceptible individuals (*S*), exposed but not yet infected individuals (*E*), infectious and symptomatic individuals (*I*), asymptomatic individuals (*A*) which means infectious but not symptomatic and recovery individuals (*R*). The model began with the state of susceptible individuals (*S*), and then entered the incubation state (*E*) when infected. The subsequent transitions included state of infection with symptomatic (*I*) and asymptomatic state (*A*), both of them would end up with recovery state (*R*). Besides, all the states above had specific death rates, and newborns joined the susceptible individuals (*S*) with a specific birth ratio. The model diagram is shown in Fig. 1.

The SEIAR model used in this study was based on the following assumptions:

1. The model assumed that HFMD cannot propagate vertically, so the new individuals born in all kinds of people are susceptible. Set the birth rate to *br*, and the natural mortality rate to *dr*. The mortality rate of infectious individual *f* was obtained by references, only 0.03%. Therefore, it is known that the mortality rate of all kinds of people in the disease spectrum is low, and the mortality rate of population attributable to HFMD is even lower. So the mortality rate of the whole population was approximately replaced by the mortality rate of several types of people except infectious individual, whose mortality rate was set as the sum of the mortality of the whole population and the mortality of HFMD.
2. Transmission of HFMD occurs via person–person, and the transmissibility between infectious individual and asymptomatic one may be different. So, the *k* was defined as the relative transmissibility rate of asymptomatic to symptomatic individuals. At the same time, we assumed the susceptible individuals will be potentially infectious (will be infected) as long as they are in contact with infectious individuals or asymptomatic individuals, and the coefficient of the infection rate was set as *β.*
3. We considered that there was a certain proportion of exposed individuals *pE* (0⩽*p*⩽1) transformed into infectious individuals *I* after incubation, another part of exposed individuals (1 − *p*) *E* were transformed into asymptomatic individuals after incubation as well. At time *t*, the development speed from the *E* to *I* pathway is same as the *E* to *A* pathway and both of them were proportional to the number of exposed individuals. The proportional coefficient of the former was *pω*, and the latter was (1 − *p*) *ω.*
4. Infectious and asymptomatic individuals may move to *R*, with speed of recovering being in direct proportion to the number of individuals. The proportional coefficients were *γ* and *γ*′ respectively.
5. Infectious and asymptomatic individuals can produce antibodies after recovery. This study assumed that the recovered individuals have permanent immunity which means they would no longer be infected, so *R* was set as the end of the model.
6. Perinatal transmission of enteroviruses is clearly documented [14, 15]. The main modes of perinatal transmission are by intrapartum exposure to maternal blood and/or genital secretions, as well as the faecal–oral and respiratory routes after delivery [16]. However, evidence of vertical transmission of enteroviruses associated with HFMD has not been confirmed by specific studies. Serological data on enterovirus infections during pregnancy were also lacking, because it's hard to diagnose enterovirus infection in adult [17]. So we assumed that HFMD cannot propagate vertically.

The differential equations of the model were used to describe the dynamic changes of each state. The corresponding model equations are as follows:
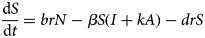

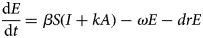

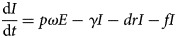

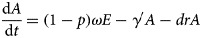

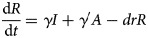


The left side of the equations represents the instantaneous velocities of *S*, *E*, *I*, *A* and *R* at time *t*.

### The seasonality of the transmission

In this study, the seasonality of the transmission was considered. According to the mechanism of the SEIAR model we built, the seasonality should be dynamic, focusing on *β*. Therefore, a trigonometric function was adopted and shown as follows:
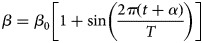


In the equation, *β*_0_, *t*, *α* and *T* refer to the baseline of the transmission relative rate, time, a constant which adjusts the position of time, and the time span of the season cycle respectively.

### Estimation of parameters

Of *p*, *ω*, *γ* and *f*, the four parameters in the model (Table 1) came from the literatures. The range of the symptomatic infection ratio was from 19% to 47% [2, 18, 19], which was chosen as the median 44.23%, that is, *P* = 0.4423. The range of incubation *1/ω* was from 3 to 7 days [2, 20], with 5 days chosen as median (with a median of 5 days), that is, *ω* = 0.2. The course of symptomatic infection was 2 weeks [21, 22], that is, recovery rate of the infection *γ* = 0.0714. The range of course of asymptomatic infection *1/γ*′ was 2 to 4 weeks [3, 18, 19], with 3 weeks chosen as median (with a median of 3 weeks), that is, the recovery rate of the asymptomatic *γ*′ = 0.0476. The range of the fatality rate was from 0.0001 to 0.0005 [23-25], with 0.0003 chosen as median (with a median of 5 days). The parameters *β* were estimated by curve fitting. There was no clearly relevant data or reference to support the parameter *k* which still remained uncertain. Thus, *k* = 1 was selected for calculation, and sensitivity analysis was carried out to figure its influence on the model. The birth rate (*br)* and the mortality rate (*dr)* were collected from Xiamen Centre for Disease Control and Prevention.

**Table 1.** Parameter definitions and values

Parameter	Description	Unit	Value	Method
*β*	Transmission relative rate	(individual × day)^−1^	–	Curve fitting
*k*	Relative transmissibility rate of asymptomatic to symptomatic individuals	1	1	–
*p*	Proportion of the symptomatic	1	0.4423	References [2, 18, 19]
*ω*	Incubation relative rate	Day^−1^	1/5	References [2, 20]
*γ*	Recovery rate of the infectious	Day^−1^	1/14	References [21, 22]
*γ′*	Recovery rate of the asymptomatic	Day^−1^	1/21	References [3, 18, 19]
*f*	Fatality rate of HFMD cases	1	0.03%	References [23–25]
*br*	Birth rate	1	0.0089747	From Xiamen CDC
*dr*	Natural death rate of the whole city	1	0.004529	From Xiamen CDC

CDC: Center for Disease Control and Prevention.

### Transmissibility of HFMD

In this study, the effective reproduction number *R*_eff_ was used to measure the transmissibility of HFMD, thought of as the number of new cases which was produced by a typical case during the period of infection. If it is more than 1, it indicates that the transmissibility of the disease is strong and the disease will be able to spread in the population and the opposite is true when the effective reproduction number is less than 1.
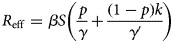


### Sensitivity analysis

There was no clearly relevant data or literature to support the parameter *k* which is still uncertain. Therefore, the sensitivity was tested by dividing *k* into six values within the range of 0 to 1, that is, *k* = 0, *k* = 0. 2, *k* = 0. 4, *k* = 0. 6, *k* = 0. 8 and *k* = 1 (indicates transmissibility rate of asymptomatic individuals is the same as symptomatic ones).

### Simulation methods and statistical analysis

In this study, Berkeley Madonna 8.3.18 (developed by Robert Macey and George Oster of the University of California at Berkeley. Copyright ©1993–2001 Robert I. Macey & George F. Oster) was employed for fitting the model with the data to calculate the parameters, while the transmissibility was calculated based on the parameters. The determination coefficient (*R*^2^) was used to evaluate the goodness of fit of the model simulation. The regression analysis, which was used to calculate *R*^2^, was performed by the software SPSS 21.0.0.0 (IBM Corp., Armonk, NY, USA).

## Results

### Incidence of HFMD in Xiamen

**Fig. 2.**
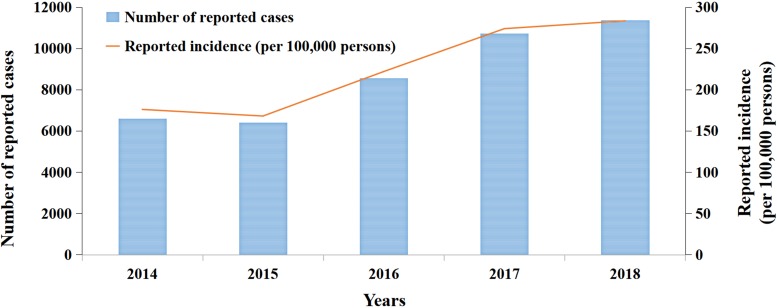
Number of reported cases and incidence of HFMD in Xiamen City from 2014 to 2018.

A total of 43 659 cases of HFMD were reported in Xiamen for the period 2014 to 2018 (Fig. 2). The median annual reported incidence was 221.87 per 100 000 persons (range: 167.98 per 100 000 persons–283.34 per 100 000 persons). The number of reported cases and the incidence showed an upward trend over the past 5 years except in 2015, which was shown to have a slight decrease. The upward trend was the most obvious between 2015 and 2017, and slowed down after 2017.

### Simulation results of HFMD transmission by the SEIAR model

**Fig. 3.**
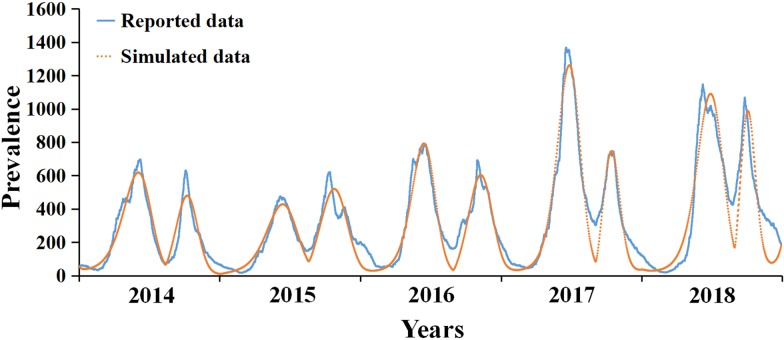
Curve fitting results based on the data in Xiamen, 2014–2018.

The results of curve fitting between the model and the reported cases are illustrated in Fig. 3. It was found that there are two epidemic cycles in summer and autumn each year, whose peaks were in June and October, through the analysis of the reported cases and curve of fitting.

As was depicted in the result of curve fitting, it was shown that the number of cases had a periodic increase trend except in 2015, which had a slight decrease. After 2015, the increasing speed of reported cases was apparent in 2017 and 2018, whose number of reported cases increased obviously than the previous 3 years.

Table 2 lists the fitting values of *β*_0_, *R*^2^ and *P* based on the data from 2014 to 2015. The mean of *β*_0_ is 2.9270 × 10^−8^, which means that the model has a great fitting effect with the data of HFMD (*R*^2^ = 0.9212, *P* < 0.0001).

**Table 2.** Estimated values of *β*_0_ and goodness of fit of the model to reported HFMD data from 2014 to 2018 in Xiamen

Year	Mean of *β*_0_	*R*^2^	*P*
2014	1.7706 × 10^−8^	0.9198	<0.0001
2015	1.5692 × 10^−8^	0.8927	<0.0001
2016	1.8454 × 10^−8^	0.9361	<0.0001
2017	3.9903 × 10^−8^	0.9572	<0.0001
2018	5.4595 × 10^−8^	0.9301	<0.0001
Total	2.9270 × 10^−8^	0.9212	<0.0001

### The transmissibility of HFMD

The median of *R*_eff_ of HFMD in Xiamen from 2014 to 2018 was 1.1048 (range: 1.4045 × 10^−5^–3.7459). The difference was significant in each year, showing obvious periodic characteristic. There were two transmission peaks each year (from April to May and August to September), and the transmissibility decreased rapidly then. During the peak period, the transmissibility of HFMD can reach 200 000 times more than the lowest. Table 3 shows the details.

**Table 3.** Effective reproduction number (*R*_eff_)

Year	The median of *R*_eff_	Maximum	Minimum
2014	1.17	2.39	0.00
2015	1.26	2.18	0.00
2016	1.12	2.64	0.00
2017	0.88	3.54	0.00
2018	1.18	3.75	0.00
Total	1.15	3.75	0.00

**Fig. 4.**
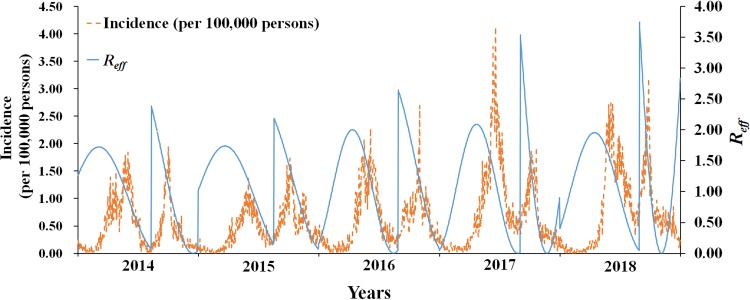
The daily incidence rate and *R*_eff_ in Xiamen, 2014–2018.

As illustrated in Fig. 4, *R*_eff_ was consistent with the incidence data, whose peaks were mostly 1–2 months before the peaks of the incidence. Both *R*_eff_ and incidence revealed the obvious periodic characteristic, with two transmission peaks each year.

### Sensitivity analysis

**Fig. 5.**
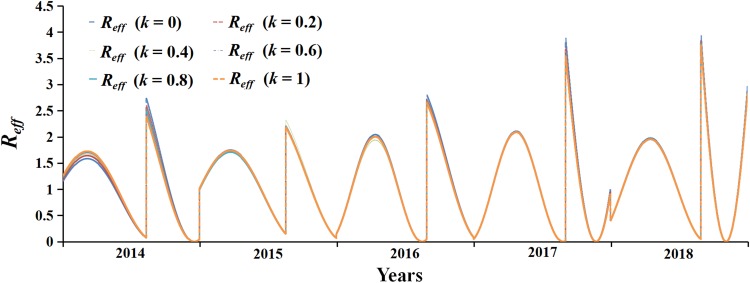
Sensitivity analysis of *k* (range: 0–1).

The fitting curve with six values of parameter *k* in the range of 0–1 had high coincidence degree, which indicates that the SEIAR model used in this study was not sensitive to it. The specific results are elucidated in Fig. 5.

## Discussion

In this study, we applied the SEIAR model with seasonal correction for the first time to clarify the transmissibility of HFMD, which would offer a guideline for the study of transmissibility, prevention and control measures of HFMD.

### Validity of model

The following three points guaranteed the validity of the model: the fitting effect of the model was tested by regression analysis (*R*^2^ = 0.9212). At the same time, sensitivity was examined by parameter *k*, which indicates that the SEIAR model used in this study is not sensitive to it. Besides, the model was based on daily data, which increase the sample and accuracy of the study.

Not only was the above enough to guarantee the validity of the model, but also ensure the reliability of the research, which paves the way for further research.

### The epidemiological characteristics of HFMD

Our finding showed that the number of HFMD cases increased in Xiamen from 2014 to 2018, except 2015. The decrease in 2015 might be due to the launch of the vaccine or the decreasing of annual rainfall (1409 mm, 289 mm less than the usual, data from Xiamen Meteorological Bureau). The incidence increased remarkably from 2015 to 2017 and slightly from 2017 to 2018. The reason of the incidence changing remains unclear and more research studies are needed to explore the mechanism of the transmission.

Obvious seasonality and the two peaks twice a year of HFMD had been shown in the study, which were consistent with the previous studies [26–29], and the conclusion on time distribution of cases in summer and autumn also fits basically the previous studies [28, 30]. However, it can be found that there was no significant difference between the two incidence peaks, which was different from the point of view of Zhang *et al*. [28].

### Assessment of transmissibility of HFMD

The transmissibility of HFMD varies greatly within a year, the highest peak value was 200 000 times more than the lowest, and the annual median of *R*_eff_ was 2.2–3.7, which indicated that the transmission rate of HFMD in Xiamen is high that will result in the increase in the number of patients with HFMD. The range of *R*_eff_ in previous studies was obviously disparate because of different experimental designs. Such as 1.14–1.24 by Yang *et al*. [31], which was slightly lower than this study, it might be influenced by its focus that placed more emphasis on community. The range of *R*_eff_ was between 2.5 to 5.5 from two studies in Hong Kong and Singapore [32, 33], which was approximately consistent with this study.

The seasonal characteristics of the transmissibility of HFMD are notable. Its first and second peaks are from March to April and August to September every year. The transmissibility during the other months is relatively low. The peak of transmission in autumn was stronger than in spring. Some studies showed that CA-V16 might lead to the peak of HFMD in autumn [34]. The intensity difference between the peaks of HFMD in Xiamen may be related to the etiological characteristics of HFMD.

The periodicity of *R*_eff_ is earlier than the incidence of HFMD, and its peak is 1–2 months earlier than the latter, which indicates that the transmissibility of HFMD is beforehand the outbreak of HFMD and offers some indication for the prevention and control of HFMD.

### The significance for the disease control

At present, the main measures to prevent and control HFMD are by enterovirus vaccine, as well as the early identification and intervention of disease outbreaks [3]. According to the conclusion of this study, the identification time of epidemic situation should be adjusted to the time before the peak of incidence, that is, the peak of *R*_eff_, in order to accord with the epidemiological characteristics of the disease, and to prevent and control it earlier, whose specific time should be 1–2 months ahead of the peak of incidence.

Greater effect will be received if the correct time of intervention is chosen ahead of the peak in transmissibility of HFMD. The measures should be applied to the susceptible population, that is, from February to March, July to August of each year, so as to control the spread of epidemic situation to the maximum extent and get the greatest prevention and control effect. For example, the school term for the children under the age of 10 years should be changed to later dates in the areas with a high incidence of HFMD, to cause a change in the peak and interfere with the spread of infection.

The results of this study also have guiding significance for vaccination. The previous study showed that the durability of neutralising antibody induced by EV-A71 vaccine was limited, and it was necessary to strengthen the injection for long-term protection [35]. The seasonal transmissibility of HFMD should be considered into vaccine injection time and strengthening injection time, which can be placed in February and July of each year, to increase the income of vaccine and achieve more effective prevention and control.

### Limitations

Limited by the availability of data, there are some limitations in this study. Some factors (gender and age) which might affect the transmissibility of the disease were not considered in the model. In addition, there may be some difference in the fitting of the epidemic situation and the estimation of the transmissibility in other high-latitude areas, as this study is confined to Xiamen. Besides, for the reinfection of HFMD, there could be a 2.02% probability according to the previous literature [36]. Considering that the scale of the proportion, the maintenance time of the antibody is greatly affected by various factors as well. Therefore, this study abandoned pathway of recovered/removed individuals transforming into susceptible individuals when establishing the model.

The vertical transmission of HFMD was not considered, which might slightly impact the result of our study.

## Conclusions

Based on the incidence data of Xiamen from 2014 to 2018, the SEIAR model has a great fitting effect in the incidence situation of HFMD. It's found that the incidence and transmissibility of HFMD are seasonal, which have two seasonal peaks. The peak of incidence is in summer and autumn every year, and the peak of transmissibility is 1–2 months ahead of the peak of incidence.

## Data

Extra data is available by emailing to Dr Mingzhai Wang (xmcdcwmz@qq.com) on reasonable request.
